# How technology-enhanced experiential e-learning can facilitate the development of person-centred communication skills online for health-care students: a qualitative study

**DOI:** 10.1186/s12909-022-03127-x

**Published:** 2022-01-25

**Authors:** Faith Liao, David Murphy, Jeng-Cheng Wu, Chien-Yu Chen, Chun-Chao Chang, Po-Fang Tsai

**Affiliations:** 1 Department of Medical Education, Taipei Medical University Hospital, 252 Wu-Xing Street, Xinyi District, 110 Taipei, Taiwan; 2 Graduate Institute of Humanities in Medicine, College of Humanities and Social Sciences, Taipei Medical University, 250 Wu-Xing Street, Xinyi District, 110 Taipei, Taiwan; 3 School of Education, University of Nottingham, Jubilee Campus, Wollaton Road, Nottingham, NG8 1BB UK; 4 Department of Urology, Taipei Medical University Hospital, 252 Wu-Xing Street, Xinyi District, 110 Taipei, Taiwan; 5 Department of Urology, School of Medicine, College of Medicine, Taipei Medical University, 250 Wu-Xing Street, Xinyi District, 110 Taipei, Taiwan; 6 Department of Anesthesiology, Taipei Medical University Hospital, 252 Wu-Xing Street, Xinyi District, 110 Taipei, Taiwan; 7 Department of Anesthesiology, School of Medicine, College of Medicine, Taipei Medical University, 250 Wu-Xing Street, Xinyi District, 110 Taipei, Taiwan; 8 Division of Gastroenterology and Hepatology, Department of Internal Medicine, Taipei Medical University Hospital, 252 Wu-Xing Street, Xinyi District, 110 Taipei, Taiwan; 9 Division of Gastroenterology and Hepatology, Department of Internal Medicine, School of Medicine, College of Medicine, Taipei Medical University, 250 Wu-Xing Street, Xinyi District, 110 Taipei, Taiwan

**Keywords:** Medical education, Communication skills, E-learning, COVID-19, Online learning, Person-centred approach, Qualitative study

## Abstract

**Background:**

The COVID-19 pandemic brought a new challenge to medical education—health-care students had fewer opportunities to interact with and treat real patients in clinical settings. Interpersonal communication skills are often developed through human interaction and communication in person, and few studies have proposed feasible digital solutions to develop learners’ communication skills. Consequently, understanding how medical teachers facilitate and implement online training programmes, with feasible instruments, to enhance students’ learning effectiveness when in-person training is not possible is critical.

**Methods:**

By using a convenience sampling method, we recruited 26 health-care students from seven medical schools in Taiwan. Through semistructured interviews and the thematic analysis technique, we analysed the latent learning factors from the experience of implementing the technology-enhanced experiential e-learning tool ‘mPath’.

**Results:**

Three themes were generated: A) *transferring theory into practice*, B) *increasing authenticity with analytical features,* and C) *maintaining autonomy with nondirective learning*. The features *accessibility, flexibility, intractability, and visualisation* with the characteristics of *remote accessibility and flexibility*, *repetition and retrospect*, *feedback requesting,* and *visualised analytical reports* were considered to enhance learning outcomes.

**Conclusion:**

This study indicated *how* online training using technology could develop the participants’ person-centred communication skills and what features influenced the learning outcomes of social distancing. mPath may be a feasible online learning approach and has provided inspiration for developing health-care students’ communication skills when in-person training is not possible.

## Background

A beneficial therapeutic relationship, achieved through the application of adequate levels of Rogerian (1957) attitudinal relationship conditions, expressed through person-centred communicational skills, is a well-known factor influencing patient outcomes [1–3]. The original person-centred approach is grounded in the work of Carl Rogers, an American psychologist who first developed client-centred therapy. From these origins in client-centred therapy, the person-centred approach emerged as the wider application of the theory and philosophy to many settings, professional contexts and as a philosophy of life detailed in Rogers’ (1980/1995) book *A Way of Being* [4]. Rogers’ theory is the most extensively researched and theoretically rigorous understanding of person-centred relationships, with over 70 years of empirical research findings on the effectiveness of person-centred relationship conditions in leading to positive therapeutic outcomes [5]. In addition, as Rogers’ theory of a person-centred approach is grounded in a philosophically sound ontological foundation of the concept of a ‘person’, the theory that follows has a high degree of internal theoretical consistency and coherence. This means that there is both philosophical and theoretical bases for practice and application. Rogers’ approach answers both ‘how’ and ‘why’ such an approach can be adopted. By contrast, other person-centred approaches do not specifically draw from philosophical and theoretical foundations to the same degree of rigour [6–8].

Since the COVID-19 pandemic hit in 2020, the teaching and learning approaches of communication skills in medical and healthcare worker’s education have been unexpectedly influenced and limited due to spatial restriction. For example, prehealth-care students have had fewer opportunities to encounter patients at the beginning of the outbreak and were even requested to withdraw from the early clinical exposure (ECE) programme [6], one of the most effective learning approaches to develop interpersonal and communication skills. Many medical and healthcare educators were advised to continually support students’ learning *without* arranging any direct patient care activities in the course as an alternative [7]. The withdrawal has called for the adaptation of current training methods to fulfil the goal of developing health professionals with adequate skills and experience [8]. Hence, modifying the methods of communication skill training in medical education has become crucial.

Historically, medical and healthcare education is like a living creation evolving to deliver a combination of knowledge, intrapersonal, and interpersonal skills to learners with flexibility, adaptabilities, and capacities [9]. Various pedagogies in medical education have been applied constructively in achieving applicability in clinical practice and advancing the quality of healthcare [10–13], such as Miller’s pyramid, problem-based learning (PBL), and Kolb’s (1984) experiential learning, and all have been effectively adapted and enhanced students’ learning effectiveness in transforming medical knowledge to clinical practice [8, 10, 14–17]. Rogers and Barrett-Lennard also emphasised that facilitating a specific learning environment can enhance the trainees’ personal and training experience in learning [2, 8, 18]. Medical educators should develop effective courses for learning communicational skills online, which would successfully maintain and enhance the learning outcome for health-care students [19, 20]. Liao and Murphy reported continual growth of communication skills ability, with skills abilities continuing to develop after the class-based training with lectures, group discussions, and the use of online technology had ended [18].

Online teaching and learning, namely contemporary e-learning and e-teaching, has played an essential role in medical education worldwide by meeting multiple expectations in training, such as medical knowledge acquisition and exchange, cognitive skills development by simulating real-world practice [21–25], and finally, knowledge correction and performance adjusting in time for developing higher-level cognitive abilities. However, e-learning seems to provide insufficient opportunities for interpersonal interaction between peers and teachers, while the ECE programme is not available to health-care students. An approach to e-learning that can offer specific features to evaluate learners’ learning process and outcomes is therefore required [22].

The pandemic has challenged the degree of adaptability and creativity of medical teachers who are expected to be role models, facilitators, assessors, planners, resource developers, and information providers [26, 27] while attempting to cover all elements of the curriculum, including communication skills. *Carl Rogers’ Freedom to Learn (1983)* and *Hill’s Helping Skills Model* (2009) have previously addressed the theories of teaching and learning for medical educators in learning communication skills [18, 28, 29]. For example, the ideal environment for communication skills training, including the use of learning tools and instruments, emphasises facilitating an experiential learning environment, initiating self-directed learning, and enhancing self-reflection [1, 18, 30–34]. In the context of a pandemic and the shift of learning into the online environment, e-learning tools that can support the needs of medical and healthcare educators are required. Some studies have indicated that e-learning tools with features that allow learners to make annotation notes embedded on recorded practice videos; adapt to review different perspectives of interactions; request direct peer, tutor, or patient feedback; deepen understanding of the effect; and observe body movements to help reflect on various facets of students’ experiences during practice interactions with peers can effectively improve learning outcomes. One such system that offers all of the above is mPath learning software [18, 31, 35].

Therefore, as healthcare educators strive to expeditiously adopt online teaching and learning approaches [27] for medical and healthcare students, in this study, we analysed data from Liao and Murphy (2019) to further our understanding of *how* learners develop person-centred communication skills when using e-learning technology such as mPath. More specifically, we explored *how* they worked with the software interface and *what* possible latent factors were responsible for effecting learning when in-person training is not possible.

## Methods

### Study objectives

We explored Liao and Murphy’s report on the evaluation of the therapeutic relationship communication skills training, which innovatively consisted of five class sessions including a combination of didactic lectures, conversational-simulation experiential exercises, and nondirective tutor facilitated reflection and individual self-reflection processes with the use of mPath [21]. In the report, the participants were spread across five specialties who shared the same level of baseline competence in communication skills [*F* (4, 54) = .28, *p =* .887], and no significant differences in competence were noted between the control (*N* = 33) and experimental (*N* = 26) groups [*t*(57) = .38, *p =* .71] prior to the training [21]. After the training, results revealed a significant change in therapeutic relationship competence for the experimental group attending the workshop (MD = + 9.5, *p =* .002) and presented a continuing growth (MD = + 19.423, *p =* .000) 2 weeks after the intervention while the control group exhibited a decline (MD = −.515, *p =* .812) [21]. Semistructured interviews were conducted to understand to what extent learners’ learning and the experience of changing skills had occurred in response to the innovative pedagogy incorporating mPath, as described in the previous study [21].

mPath is designed for developing learners’ interpersonal skills during off-training moments. It allows learners to gather their annotations and affective responses to video-recorded skills practice sessions in either a textual form or a graphic format, facilitating self-reflection, and to request and respond to peer feedback on the recorded practice sessions [20, 21, 34, 36]. However, how the participants develop skills with mPath and which features enhance learns knowledge remain unexplored. Therefore, to overcome the impact of COVID-19 in medical education in severely curtailing in-person training, we explored the use of mPath as a possible means of online communication skills training. We examined the features and functions of this technology-enhanced experiential e-learning instrument to achieve teaching goals for interpersonal skill training in the era of social distancing.

### Study design

This qualitative study analysed data collected through semistructured interviews with 26 Taiwanese health-care students. The interviews aimed to understand the participants’ experience of learning person-centred communication skills during training, specifically through the use of a technology-enhanced pedagogy. This analysis was intended to further explore the scope for the plausible factors influencing their development of interpersonal competences. The semistructured interview questions were classed into three categories as presented in Table 1.

**Table 1:** Semi-structured Interviewing Questions

Questions	Content
1	What did you consider helpful in regard to your learning experience using the mPath? Please elaborate.
2	How did you maintain/develop the competences during and after the intervention? What was the reason you might think of? Please elaborate.
3	How did you find the technology-enhanced experiential e-learning with mPath in the communication skills training? Please elaborate.

### Participants

In this study, 26 interviewees (2 men and 24 women) were taken from the experimental group of the study by Liao and Murphy [21]. They were recruited through convenience sampling and consented to participate in the study. The group comprised 15 participants specialising in nursing, 5 in occupational therapy, 4 in medicine, and 2 in clinical psychology. Most participants were 20–29 years old, women, and had experienced early clinical exposure (ECE) as clerks or interns in clinical settings. Table 2 summarises the interviewees’ demographic characteristics.

**Table 2:** Demographic characteristics of participants

Characteristic	N	%		N	%
**Age (years)**			**Speciality**		
*20–29*	24	92.3	*Nursing*	15	57.7
*30–39*	1	3.85	*Occupational Therapy*	5	19.2
*40–49*	1	3.85	*Medicine*	4	15.4
*> 50*	0	0	*Clinical Psychology*	2	7.7
*Means (M)*	22.96 (years)				
*Standard Deviations (SD)*	5.64				
**Gender**			**Experienced Early Clinical Exposure**		% of each speciality
*Male*	2	7.7	*Nursing*	14	93.33
*Female*	24	92.3	*Occupational Therapy*	2	40
			*Medicine*	4	100
			*Clinical Psychology*	0	0
			*Total*	20	76.92

### Data collection

A combination of the semistructured interview allowed the researcher to collect the information and encouraged the participants to communicate, discuss, and comment on each other’s experiences and perspectives openly and help them reflect on the learning experience [1, 37]. Twenty-four interviewees were interviewed in person and two through a Skype conference call, with each interview lasting approximately 45 min to 1 h. The semi-structured interviews were conducted after the technology-enhanced training workshop, one or two interviewees at one time. Although we might collectively gather individual view, this would not be a serious focus-group method. The researcher did not play the role as moderator, but conducted interview according to the semi-structured outline. However, considering the fact that most participants were interviewed just after the same workshop, it would be natural situation that they might discuss each other’s experience. Even if it was unexpected, we believed that information was also valuable for our research.

### Thematic analysis

We applied Braun and Clarke’s (2006) six phases of thematic analysis. All interviews were audio recorded and transcribed verbatim. The transcriptions were confirmed with the interviewees to ensure reliability and remained faithful to the original information. Each transcription was read thoroughly and repeatedly to generate the codes across the interviews. The coded data were categorised as themes to capture the sense of significance, and they were also discussed and explored to evaluate whether they purposefully referred to the unexpected finding in the study. The initial themes were re-examined and the coding framework was refined to generate a satisfactory thematic map of the study. Before presenting the final report, the final thematic map was checked for coherence with the conceptual framework of the research and whether it consistently reflected the content of the data [38].

## Results

We observed successful outcomes with respect to the participants’ learning experience through technology-enhanced experiential e-learning by using mPath. By following Braun and Clarke’s (2006) six phases of thematic analysis, the transcriptions were coded, re-examined, and refined into three main themes in the thematic map: A) *transferring theory into practice*, B) *increasing authenticity with analytical features,* and C) *maintaining autonomy with nondirective learning*; these themes were addressed in the learning experience with mPath. The features *accessibility, flexibility, intractability, and visualisation* with the characteristics *remote accessibility and flexibility*, *repetition and retrospect*, *feedback requesting,* and *visualised analytical reports* were considered to yield enhanced learning outcomes. The participants were satisfied with the use of mPath in developing person-centred communication skills and reported an increased understanding towards the theory of person-centred communication skills. This was performing communication skills and increasing the quality of therapeutic relationship [36]*.*

### Transforming theory into practice

Six participants reported that they had learnt about Carl Rogers’ person-centred approach and communication skills in the university but had few opportunities to apply it in practice. *Transforming theory into practice* was considered a main factor that influenced the increasing in their level of therapeutic relationship competency when engaging with technology-enhanced experiential e-learning using mPath. For example:

*Participant C1: We barely have opportunities to practice any theory we have learnt in the clinical psychological courses. Many theories were given in one module but not many practical sessions for us to learn more about person-centred communication skills. (Clinical Psychology, female, 21 y/o, no experienced early clinical exposure)*

*Participant B4: Using mPath had given me opportunities to experience how the communication skills could be delivered outside of the placement. Previously, we could only apply the skills while having the placement in the hospital and the knowledge of theory could not be put into practice often. (Occupational therapy, female, 20 y/o, experienced early clinical exposure)*

### Increasing authenticity with analytical features

Participants reported an increase in their authenticity through technology-enhanced experiential e-learning online training. The objective aspects of the participants were gained towards their psychological states and behaviours. For example, the participants engaged in in-depth reflection on their body language and facial expression and pondered on their possible response to certain foreseeable situations when conducting conversations with clients in the future.

*Participant C5: mPath helped me to be more aware of my body language. And also, I start to observe my friends’ body language while they were talking. I kept asking myself why I have those movements and what they represent? I reflect on it a lot now. It gets clearer if I am in a third-person perspective. (Medicine, male, 23 y/o, experienced early clinical exposure)*

**Fig. 1:**
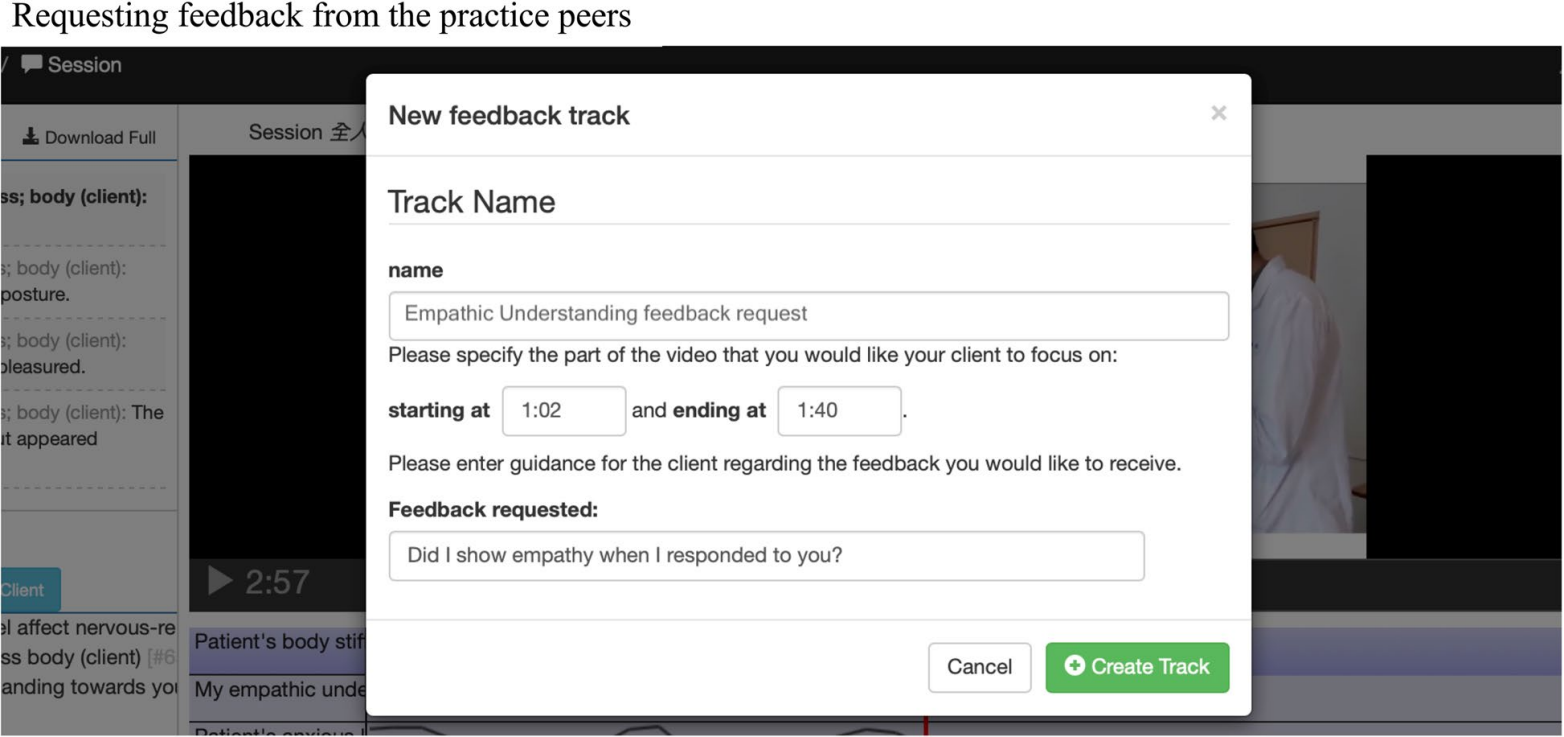
Requesting feedback from the practice peers

mPath allowed the participants to notice the details and missing information in the practice sessions. Repeatedly watching the recording sessions enhanced their in-depth self-reflection from an objective perspective compared with a subjective opinion from others (Fig. 1).

*Participant A7: There were so many details in one practice session. mPath helped me to watch the recording videos multiple times. I was not a fan of my voice, so I tried hard to get the best out of my recordings. In the end, I found it helpful when I watched it repeatedly because I could then map in my mind and ask myself, “what if it happened during my placement?” and “what kind of response I could take next time?” (Nursing, female, 21 y/o, experienced early clinical exposure)*

**Fig. 2:**
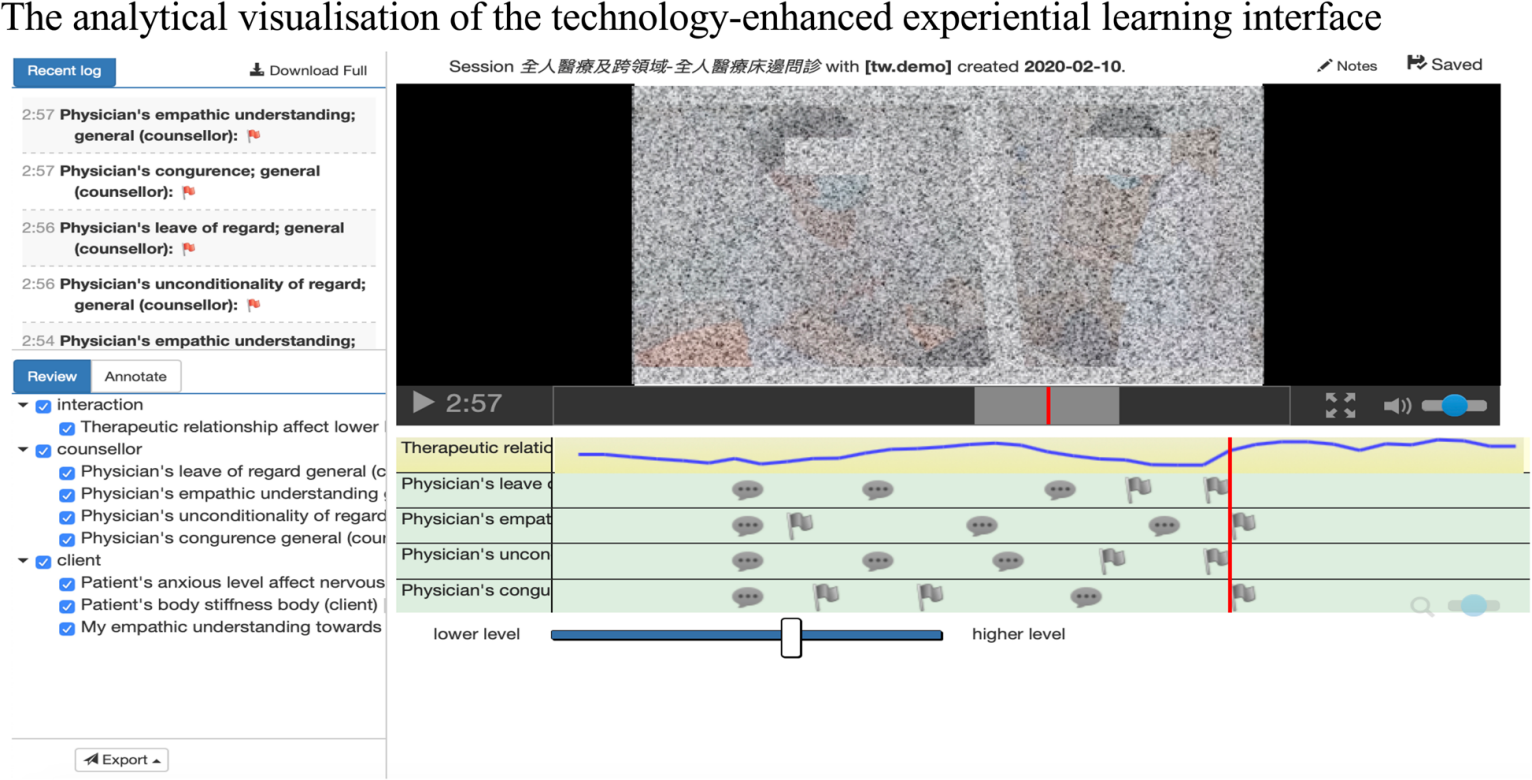
The analytical visualisation of the technology-enhanced experiential learning interface”

The analytical visualisation of mPath grouped a wide range of information (Fig. 2), for example, the categorised annotation on the tracks, recording of ratings using the *affect slider* function, and analytical logs [30]. The participants reported that the presentation in the interface helped them gain a deeper understanding of themselves regarding the specific issues.

*Participant C6: I didn’t notice that I smirked when I heard of a sad story of patients until I saw my recording in mPath. I found that I am not showing what I feel at all. I couldn’t even sense my feelings and express them directly to my close friends and family (Medicine, female, 23 y/o, experienced early clinical exposure)*

### Maintaining autonomy with nondirective learning

Autonomy was maintained when the participants could freely explore themselves when using mPath, thereby increasing the positive experience of learning person-centred communication skills. One of the participants found herself to be unknowingly influenced by social stigma during the in-person practice. She stated the following after reflecting on her therapist–patient relationships:

*Participant C6: I have revisited the recording a lot. After learning online, I have the chances and time to tune my manners in the online session and have learnt to listen to them and understand their values as human beings. (Medicine, female, 25 y/o, experienced early clinical exposure)*

Maintaining autonomy during the online session influenced the participants’ person-centred communication skills not only during the online training but also during their daily practice. The participants were allowed to revisit the recordings without restrictions. This may explain the continuous growth of the therapeutic relationship competence and communication skills without exposure to the intervention environment in the previous study [18].

## Discussion

Technology-enhanced learning has been applied to improve clinical reasoning and physical examination by using games and virtual patients [30]. Few studies have investigated the proposal of feasible digital instruments to develop learners’ soft skills when training for online communication skills. Rogers (1965) and Liao and Murphy (2019) suggested that facilitating a suitable learning environment could enhance the learners’ learning experience. Offering learners a nondirective curriculum in a culture of trust, nonjudgemental atmosphere, honesty, and participation nurtures and enhances engagement and self-directive learning [18, 30, 35].

In this study, we investigated how technology-enhanced experiential e-learning with mPath can serve as a communication skill training instrument during social distancing, such as during the COVID-19 pandemic. Thus, contemporary e-learning and e-teaching in medical education can go beyond merely playing the role of knowledge acquisition and exchange. The technology-enhanced experiential e-learning offered in this study deepened the participants’ reflection, communication, autonomy, and authenticity.

The result not only echoed the scholars’ statement of enhancing the learning effect by providing a suitable learning environment but also evidenced the participants receiving a promising outcome with increasing autonomy and authenticity. Moreover, it indicated that the features *accessibility, flexibility, intractability, and visualisation* would significantly affect learners’ online learning experience. The technological features such as 1) *remote accessibility and flexibility*, 2) *repetition and retrospect*, 3) *feedback requesting*, and 4) *visualised analytical reports* provided the participants with the opportunities to develop their person-centred communication skills and therapeutic relationship competence online [30, 31].

First, *remote accessibility and flexibility* enhanced learners’ autonomy in learning because learners could revisit the training sessions remotely without time and spatial limitations. Second, *repetition and retrospect* enabled participants to rewind and revisit the recording of the training sessions, thus increasing the authenticity of the training and allowing participants to deepen their in-depth reflection regarding their communication skills. The feature allowed the participants to self-direct and self-process to enhance their self-reflection from an objective angle. It also helped them to adapt their response from a third-person perspective [31, 33, 39]. In Chinese culture, feedback and comments are not often provided face to face. Through the *feedback requesting* feature, learners could request and receive feedback and comments from their peers virtually, thereby allowing them to overcome the cultural limitations. This also allowed users to be less resistant and defensive when learning from the recording sessions, dissect the process, and gain a deep understanding during the practice sessions [34]. Finally, through the feature *visualised analytical reports*, the interface visualised the self-analytical reports after collecting the annotations during the online training to provide notices for subverbal communication skills, such as body language, facial expression, and speaking tone [33, 40].

The four characteristics in this study not only reflected Rogers’ person-centred approach in education and highlighted the qualitative evidence of the latent factors that enhanced the effectiveness of person-centred communication skills online practice [1] but also suggested the feasibility of using the features and functions in the training instruments of online communication skills learning, especially during the COVID-19 pandemic.

This study had some limitations. First, although we conducted practising partner group interviews, we did not conduct speciality-focus group interviews, which would have enabled us to grasp more information about how nursing, medical, occupational therapy, and clinical psychology students learnt the therapeutic relationship skills before, during, and after the intervention. Second, health-care specialties were not evenly distributed across the cohort, with nursing students considerably outnumbering those in the other three groups. Future studies should recruit similar sizes of participants in each speciality.

## Conclusions

In times of limited in-person interaction such as the COVID-19 pandemic, establishing better communication and interpersonal interaction is crucial to advancing the quality of care. In this study, we attempted to overcome the restrictions to in-person training in medical education by providing an e-learning environment in which the specific features and particular characteristics significantly enhanced learning effectiveness. It has reflected and responded to the urge to develop communication skills in e-learning, while few studies have proposed feasible digital instruments in the past. Our findings not only demonstrate successful implementation of a technology-enhanced experiential e-learning tool to enhance learning outcomes but also indicate how health-care educators could facilitate online communication skill training to enhance the health-care students’ learning.

## Data Availability

The datasets used and/or analysed during the current study are available from the corresponding author on reasonable request.

## Abbreviations

ECE: Early Clinical Exposure
PBL: Problem-based learning

## References

[1] Rogers C (1983). Freedom to learn for the 80s.

[2] Barrett-Lennard G (2018). Experientical learning for professional helpers: a residential workshop innovation.

[3] Rogers CR (1957). The necessary and sufficient conditions of therapeutic personality change. J Consult Psychol.

[4] Rogers CR (1995). A way of being.

[5] Murphy D, Joseph S. Person-centered therapy: past, present and future orientation. In Cain DJ, Keenan K, Rubin S Humanistic Psychotherapies: Handbook of Research and Practice (pp. 185–218): Washington, DC: American Psychological Association; 2016.

[6] McCormack B, McCance TV (2006). Developing a conceptual framework for person-centred nursing. J Adv Nurs.

[7] McCormack B, Karlsson B, Dewing J, Lerdal A (2010). Exploring person-centredness: a qualitative meta-synthesis of four studies. Scand J Caring Sci.

[8] McCance T, McCormack B, Dewing J. An Exploration of Person-Centredness in Practice. OJIN: The Online Journal of Issues in Nursing. 2011;16(2):Manuscript 1. 10.3912/OJIN.Vol16No02Man01. 22088150

[9] Emanuel E (2020). The inevitable reimagining of medical education. JAMA..

[10] Association of American Medical Colleges. Association of American Medical Colleges. [Online].; 2020. Available from August 14, 2020: HYPERLINK. "https://www.aamc.org/media/43311/download".

[11] Taylor D, Hamdy H. Adult learning theories: implications for learning and teaching in medical education: AMEE Guide no. 83., Medical Teacher. 2013;35(11):e1561–72. 10.3109/0142159X.2013.828153. 10.3109/0142159X.2013.828153 24004029

[12] World Federation for Medical Education. Basic medical education WFME global standards for quality improvement. Denmark; 2003.

[13] Collins C, Nanda S, Palmer B, Mohabbat A, Schleck C, Mandrekar J, et al. A cross-sectional study of learning styles among continuing medical education participants. Med Teach. 2018. 10.1080/0142159X.2018.1464134. 10.1080/0142159X.2018.1464134 29703093

[14] Newble DI, Entwistle NJ (1986). Learning styles and approaches: implications for medical education. Med Educ.

[15] Nilsson MS, Pennbrant S, Pilhammar E, Wenestam CG. Pedagogical strategies used in clinical medical education: an observational study. BMC Med Educ. 2010;10(9). 10.1186/1472-6920-10-9. 10.1186/1472-6920-10-9 PMC2824800 20105340

[16] Witheridge A, Ferns G, Scott-Smith W (2019). Revisiting Miller’s pyramid in medical education: the gap between traditional assessment and diagnostic reasoning. Int J Med Educ.

[17] Albanese M (2000). Problem-based learning: why curricula are likely to show little effect on knowledge and clinical skills. Med Educ.

[18] Thistlethwaite JE, Davis D, Ekeocha S, Kidd JM, MacDougall C, Matthews P (2012). The effectiveness of case-based learning in health professional education. A BEME systematic review: BEME Guide no. 23. Med Teach.

[19] Kolb D (1984). Experiential learning: experience as the source of learning and development.

[20] Murphy D, Liao F, Slovák P, Holle L, Jackson D, Oliver P (2019). An evaluation of the effectiveness and acceptability of a new technology system to support psychotherapy helping skills training. Couns Psychother Res.

[21] Liao F, Murphy D (2019). Evaluation of therapeutic relationship skills training for mental health professionals: the therapeutic relationship enabling Programme (TREP). MedEdPublish..

[22] McCaffery M. Preparing for the worst: medical services at the Calgary Olympics. CMAJ. 1998;138(2) 151-153, 156. PMC1267554 3334928

[23] Sawant SP, Rizvi S (2015). Importance of early clinical exposure in learning anatomy. Schol J Appl Med Sci.

[24] Schön DA (1987). Educating the reflective practitioner: toward a new design for teaching and learning in the professions.

[25] Ellaway R, Masters K, Ellaway R, Masters K (2020). E-learning in healthcare education. E-learning in medical education. AMEE GUIDE 32: teaching and learning.

[26] Pannese L, Carlesi M (2007). Games and learning come together to maximise effectiveness: the challenge of bridging the gap. Br J Educ Technol.

[27] Begg M, Dewgurts D, Macleod H. Game-informed learning: applying computer game processes to higher education. Innovate. 2008;1(6) http://www.innovateonline.info/index.php?view=article&id=176 (accessed 24 April 2008).

[28] Ellaway R, Dewhurt D, McLeod H (2004). Evaluating a virtual learning environment in the context of its community of practice. ALT-J, Res Learn Technol.

[29] Harden RM, Crosby J (2000). AMEE Guide no 20: the good teacher is more than a lecturer - the twelve roles of the teacher. Med Teach.

[30] Dornan T, Boshuizen H, King N, Scherpbier A (2007). Experience-based learning: a model linking the processes and outcomes of medical students’ workplace learning. Med Educ.

[31] Wang C, Ng C, Brook R. Response to COVID-19 in Taiwan: big data analytics, new technology, and proactive testing. JAMA. 2020. 10.1001/jama.2020.3151. 10.1001/jama.2020.3151 32125371

[32] Harden R, Laidlaw J, Harden R, Laidlaw J (2017). Chapter 25. Indepent learning. Essential skills for a medical teachers: an introduction to teaching and learning in medicine.

[33] Holden J, Cox S, Irving G (2020). Rethinking role models in general practice. Br J Gen Pract.

[34] Murphy D, Slovák P, Thieme A, Jackson D, Olivier P, Fitzgerald G. Developing technology to enhance learning interpersonal skills in counsellor education. Br J Guid Counsel. 2017. 10.1080/03069885.2017.1377337.

[35] Whitcombe SW (2004). The potetial use of peer assised learning to develop first year students readings to engage in occupational therapy problem-basedlearning course.

[36] Slovák P, Thieme A, Murphy D, Tennent P, Olivier P, G. F. (2015). On becoming a counsellor: challenges and opportunities to support interpersonal skills training. In 18th ACM conference on Computer Supported Cooperative Work & Social Computing.

[37] Clouston T, Whitecombe S (2005). An emeraging person centred model for probelm-based learning. J Furth High Educ.

[38] Murphy D, Joseph S. Contributions from the person- centred experiential approach to the field of social pedagogy. Camb J Educ. 2018. 10.1080/0305764X.2018.1488946.

[39] Hill C (2020). Helping skills: facilitating, exploration, insight, and action helping skills: facilitating, exploration, insight. And action.

[40] Braun V, Clarke V (2006). Using thematic analysis in psychology. Qual Res Psychol.

